# Rapamycin inhibits mSin1 phosphorylation independently of mTORC1 and mTORC2

**DOI:** 10.18632/oncotarget.3006

**Published:** 2015-01-30

**Authors:** Yan Luo, Lei Liu, Yang Wu, Karnika Singh, Bing Su, Nan Zhang, Xiaowei Liu, Yangmei Shen, Shile Huang

**Affiliations:** ^1^ State Key Laboratory of Biotherapy/Collaborative Innovation Center of Biotherapy, West China Hospital, Sichuan University, Chengdu 610041, Sichuan, People's Republic of China; ^2^ Department of Biochemistry and Molecular Biology, Louisiana State University Health Sciences Center, Shreveport, LA 71130-3932, USA; ^3^ Feist-Weiller Cancer Center, Louisiana State University Health Sciences Center, Shreveport, LA 71130-3932, USA; ^4^ Department of Immunobiology, Yale University School of Medicine, New Haven, CT 06520, USA

**Keywords:** Rapamycin, mTOR, mSin1, raptor, rictor

## Abstract

Current knowledge indicates that the mammalian target of rapamycin (mTOR) functions as two complexes, mTORC1 and mTORC2, regulating cell growth, proliferation, survival, differentiation, and motility. Recently mSin1 has been identified as a critical component of mTORC2, which is essential for phosphorylation of Akt and other signaling molecules. Studies have shown that rapamycin inhibits phosphorylation of mSin1. However, the underlying mechanism is unknown. Here we found that rapamycin inhibited phosphorylation of mSin1 potently and rapidly. Expression of rapamycin-resistant mutant of mTOR (mTOR-T), but not rapamycin-resistant and kinase dead mutant of mTOR (mTOR-TE), prevented rapamycin from inhibiting mSin1 phosphorylation, suggesting that rapamycin-induced dephosphorylation of mSin1 is mTOR-dependent. Surprisingly, ectopic expression of rapamycin-resistant and constitutively active p70 S6 kinase 1 (S6K1) did not confer resistance to rapamycin-induced dephosphorylation of mSin1. Furthermore, disruption of mTORC1 and mTORC2 by silencing raptor and rictor, respectively, or downregulation of S6K1 or Akt did not induce the dephosphorylation of mSin1 as rapamycin did. However, silencing mTOR or mLST8 mimicked the effect of rapamycin, inhibiting mSin1 phosphorylation. Our findings suggest that rapamycin inhibits mSin1 phosphorylation, which is independent of mTORC1 and mTORC2, but is possibly dependent on a new mTOR complex, which at least contains mTOR and mLST8.

## INTRODUCTION

The mammalian target of rapamycin or the mechanistic target of rapamycin (mTOR), a member of the phosphoinositide-3′ kinase (PI3K)-related kinase family, lies downstream of the type I insulin-like growth factor (IGF-1) receptor-PI3K [1, 2]. Studies have demonstrated that mTOR functions at least as two complexes (mTORC1 and mTORC2) in mammalian cells [1, 2]. mTORC1 contains mTOR, mLST8 (mammalian lethal with sec-13 protein 8, also termed G-protein β-subunit-like protein, GβL), PRAS40 (proline-rich Akt substrate 40 kDa) and raptor (regulatory-associated protein of mTOR) [3–9], whereas mTORC2 consists of mTOR, mLST8, rictor (rapamycin insensitive companion of mTOR), mSin1 (mammalian stress-activated protein kinase-interacting protein 1), and protor (protein observed with rictor) [10–16]. mTORC1 is sensitive to rapamycin, growth factors, energy, amino acids, stress and redox levels, and regulates cell growth and proliferation by controlling protein synthesis, lipid synthesis, and lysosome biogenesis through mediating phosphorylation of ribosomal p70 S6 kinase 1 (S6K1) and eukaryotic initiation factor 4E (eIF4E) binding protein 1 (4E-BP1) [1, 2]. mTORC2 is only sensitive to prolonged (>24 h) rapamycin exposure in certain cases and growth factors, and regulates cell survival and cytoskeletal organization in part by regulating phosphorylation of Akt [17, 18], serum and glucocorticoid-inducible kinase 1 (SGK1) [19], protein kinase C α (PKCα) [11] and focal adhesion proteins [10, 11, 20], as well as the activity of small GTPases [10, 21, 22]. Both mTORC1 and mTORC2 interact with a negative regulator, DEPTOR (DEP domain containing mTOR-interacting protein) [23], and a positive regulator, the Tti1/Tel2 complex [24]. Though the functions of the mTOR complexes remain to be unveiled, current data indicate that mTOR plays a central role in the regulation of cell growth, proliferation, differentiation, survival, and motility, as well as angiogenesis and lymphangiogenesis [2, 25].

Mutations of *MTOR* gene have recently been found to be associated with the hyperactivation of mTOR in tumors [26–28]. High frequency of mutations of other components (such as *PTEN*, *TSC*, and *PI3K*) in mTOR signaling pathway has also been observed to link to human malignant progression and poor prognosis [2, 29, 30]. Interestingly, tumor cells with deregulated mTOR signaling are more sensitive to mTOR inhibitors, supporting that mTOR pathway is a promising target for cancer therapy. Rapamycin is the first identified inhibitor of mTOR. Unlike the new generation of ATP-competitive mTOR kinase inhibitors (such as AZD8055, INK 128, PP242, and Torin 2), rapamycin cannot directly bind to mTOR. Instead, it has to first form a complex with the cytosolic protein FK506 binding protein 12 (FKBP12) and then binds to the FKBP-binding (FRB) domain of mTOR, inhibiting certain functions of mTOR [1, 2]. While two rapamycin analogs (rapalogs), CCI-779 (Temsirolimus, Wyeth) and RAD001 (Everolimus, Novatis), have been approved by the US Food and Drug Administration (FDA) for treatment of metastatic renal cell carcinoma and advanced pancreatic neuroendocrine tumors, they only display modest anticancer efficacy in many other types of tumors [31]. It has been proposed that this is related to rapalogs activation of Akt and other survival pathways through insulin receptor substrate 1 (IRS-1)/Grb10 feedback mechanisms [32–35]. However, in fact, the effects of rapalogs on Akt are complex. It has been described that prolonged (24 h) exposure to rapamycin (100 nM) caused activation of Akt in HeLa and H460 cells, weak inhibition in HEK-293T cells, and strong inhibition in PC-3 cells, although mTORC1-mediated S6K1 phosphorylation was completely blocked in all cases [18]. Therefore, the anticancer mechanism of rapamycin (or rapalogs) remains enigmatic.

In addition to inhibition of phosphorylation of S6K1 and 4E-BP1, rapamycin has recently been found to inhibit phosphorylation of rictor [36, 37] and mSin1 as well [38, 39]. While the physiological significance of rictor or mSin1 phosphorylation is under investigation, studies have revealed that S6K1 phosphorylates rictor (Thr1135), and rapamycin inhibition of this phosphorylation is by suppressing mTOR-mediated S6K1 [37]. To date, how rapamycin inhibits phosphorylation of mSin1 remains a mystery.

In this study, we found that rapamycin inhibited mSin1 phosphorylation in an mTOR kinase activity-dependent manner. To our surprise, neither mTORC1 nor mTORC2 was involved in the regulation of mSin1 phosphorylation. However, silencing mTOR or mLST8 did mimic the effect of rapamycin, inhibiting mSin1 phosphorylation. Our results imply that rapamycin inhibits mSin1 phosphorylation possibly through targeting an unidentified third mTOR complex, which contains at least mTOR and mLST8.

## RESULTS

### Rapamycin inhibits phosphorylation of mSin1 in a concentration- and time-dependent manner

**Figure 1:**
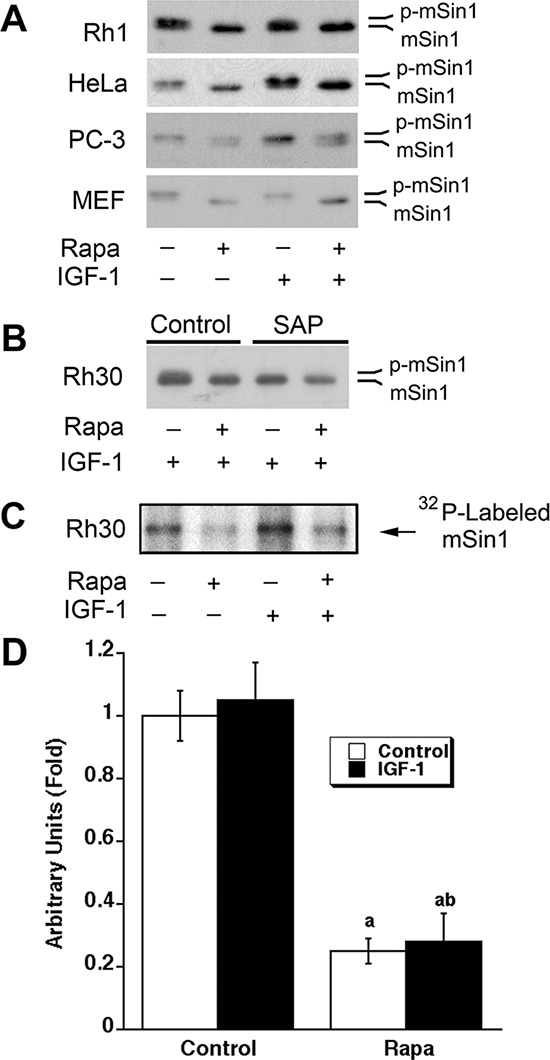
Rapamycin inhibits phosphorylation of mSin1 **(A)** Indicated cells were serum-starved for 24 h, pretreated with rapamycin (Rapa, 100 ng/ml) for 2 h, and then stimulated with or without IGF-1 (10 ng/ml) for 22 h, followed by Western blotting with mSin1 antibody. **(B)** Rh30 cells were treated as described in (A). The whole cell lysates were treated with or without shrimp alkaline phosphatase (SAP) for 30 min, and then analyzed by Western blotting with mSin1 antibody. **(C)** Rh30 cells were labeled with [^32^P] orthophosphate, followed by autoradiography, as described in “Materials and methods”. **(D)** Quantification data for panel (C). ^a^*P* < 0.05, difference vs control group; ^b^*P* < 0.05, difference vs IGF-1 group.

To determine the effect of rapamycin on phosphorylation of mSin1, Rh1 cells, a rapamycin-sensitive human Ewing sarcoma cell line [40, 41], were initially selected for the study. As there were no antibodies against phospho-mSin1 available commercially, at the beginning, we detected phosphorylation of mSin1 according to the electrophoretic mobility of mSin1 by Western blot analysis, as described by other groups [38, 39]. As shown in Figure 1A, serum starvation for 24 h did not increase the electrophoretic mobility of mSin1 in Rh1 cells, but treatment of the serum-starved cells with rapamycin (100 ng/ml) for 24 h increased the electrophoretic mobility of mSin1 obviously, regardless of stimulation with or without IGF-1 (10 ng/ml), suggesting that rapamycin might inhibit phosphorylation of mSin1. Furthermore, similar results were also observed in other cell lines, including human cervical cancer (HeLa), prostate cancer (PC-3), rhabdomyosarcoma (Rh30) cells and mouse embryonic fibroblasts (MEF) (Figure 1A and 1B), demonstrating that rapamycin inhibition of mSin1 phosphorylation is not cell line-dependent. To verify the electrophoretic mobility of mSin1 is correlated to its phosphorylation status, shrimp alkaline phosphatase (SAP) was used. We found that treatment of Rh30 cell lysates with SAP for 30 min reversed the mobility of mSin1 in the control (data not shown) or IGF-1-treated cells to that of rapamycin-treated cells (Figure 1B), revealing that the increased mSin1 mobility shift was indeed due to its dephosphorylation. In addition, rapamycin inhibition of mSin1 phosphorylation was further confirmed by ^32^P-labeling (Figure 1C, 1D).

**Figure 2:**
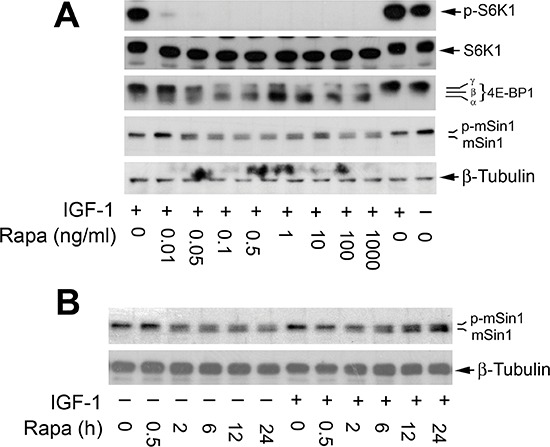
Rapamycin inhibits phosphorylation of mSin1 in a concentration and time-dependent manner Serum-starved Rh30 cells were pretreated with rapamycin for 2 h at indicated concentrations, and then stimulated with IGF-1 (10 ng/ml) for 10 h **(A)** or pretreated with 100 ng/ml rapamycin for indicated time, and then stimulated with or without IGF-1 (10 ng/ml) for 15 min **(B)**. Treated cells were harvested for Western blotting with indicated antibodies.

To better understand the inhibitory effect of rapamycin on mSin1 phosphorylation, dose-response and time-course experiments were carried out. We found that when serum-starved Rh30 cells were pre-treated with rapamycin (0–1,000 ng/ml) for 2 h, and then stimulated with or without IGF-1 (10 ng/ml) for 10 h, as expected, rapamycin inhibited the basal or IGF-1-stimulated phosphorylation of 4E-BP1 and S6K1, two best characterized downstream effector molecules of mTOR in a concentration-dependent manner (Figure 2A). Of interest, rapamycin also inhibited phosphorylation of mSin1 in a similar manner. Noticeably, rapamycin was able to increase mSin1 mobility shift at a very low concentration (0.05 ng/ml) (Figure 2A). Similarly, when the cells were pretreated with rapamycin (100 ng/ml) for 0–24 h, and then stimulated with or without IGF-1 (10 ng/ml) for 15 min, rapamycin was also found to be able to inhibit the basal or IGF-1-stimulated phosphorylation of mSin1 in a time-dependent manner. The inhibitory effect was rapid and remarkable within 2 h treatment (Figure 2B).

### Rapamycin inhibits phosphorylation of mSin1 in an mTOR kinase activity-dependent manner

**Figure 3:**
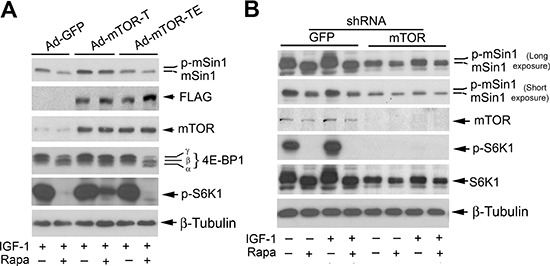
Rapamycin-induced dephosphorylation of mSin1 is dependent on mTOR kinase activity Rh30 cells, infected with recombinant adenoviruses expressing GFP (Ad-GFP), FLAG-tagged rapamycin-resistant and kinase active mTOR (S2035T, Ad-mTOR-T), and rapamycin-resistant and kinase dead mTOR (S2035T/D2357E, Ad-mTOR-TE) **(A)** or with lentiviral shRNAs to GFP and mTOR **(B)** respectively, were serum-starved for 24 h. The cells were then pretreated with or without rapamycin (Rapa, 100 ng/ml) for 2 h, and further stimulated with or without IGF-1 (10 ng/ml) for 10 h, followed by Western blotting with indicated antibodies.

It has been described that rapamycin inhibits skeletal myogenesis in an mTOR kinase activity-independent manner [42, 43], although this remains controversial [44, 45]. To determine whether rapamycin inhibition of mSin1 phosphorylation depends on the kinase activity of mTOR, serum-starved Rh30 cells were infected with recombinant adenoviruses expressing empty vector (GFP), FLAG-tagged rapamycin-resistant kinase active mTOR (S2035T, mTOR-T), and rapamycin-resistant kinase-dead mTOR (S2035T/D2357E, mTOR-TE), respectively. Subsequently, the cells were pre-treated with or without rapamycin for 2 h, and then stimulated with IGF-1 (10 ng/ml) for 10 h. Consistent with our previous observations [20, 21], expression of mTOR-T, but not mTOR-TE or GFP, potently prevented rapamycin from inhibiting phosphorylation of 4E-BP1 and S6K1 (Figure 3A), two best-characterized substrates of mTOR [1, 2]. Of note, the phosphorylation state of 4E-BP1 was detected with an antibody to 4E-BP1. Rapamycin inhibited phosphorylation of 4E-BP1, as indicated by the decrease in the intensity of the uppermost band γ and by the increase in the higher mobility band α and β that corresponds to a less phosphorylated form of 4E-BP1. The results indicate that mTOR-T functioned as a rapamycin-resistant and kinase active mutant, and mTOR-TE as a kinase-dead mutant in Rh30 cells. Interestingly, expression of mTOR-T, but not mTOR-TE or GFP, conferred high resistance to rapamycin inhibition of mSin1 phosphorylation (Figure 3A), suggesting that rapamycin inhibits phosphorylation of mSin1 in an mTOR kinase activity-dependent manner.

We also substantiated the above finding using RNA interference. As expected, silencing mTOR dramatically decreased the mTOR kinase activity, since the basal or IGF-1-stimulated phosphorylation of S6K1 (Thr389), routinely used as an indicator of mTOR kinase activity [1, 2], was almost not detectable by Western blotting (Figure 3B). Of importance, silencing mTOR remarkably reduced the basal or IGF-1-simulated phosphorylation of mSin1, even in the absence of rapamycin (Figure 3B). Collectively, our results indicate that mTOR regulates phosphorylation of mSin1.

### Rapamycin inhibits phosphorylation of mSin1 not via targeting S6K1

**Figure 4:**
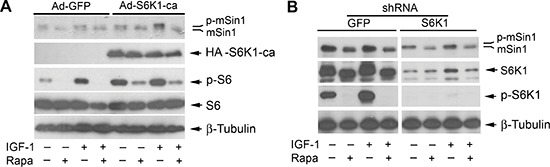
Rapamycin-induced dephosphorylation of mSin1 is not by inhibiting S6K1 Rh30 cells, infected with recombinant adenoviruses expressing GFP (Ad-GFP) and HA-tagged rapamycin-resistant and constitutively active S6K1 (Ad-S6K1-ca) **(A)** or with lentiviral shRNAs to GFP and S6K1 **(B)** respectively, were serum-starved for 24 h. The cells were then pretreated with or without rapamycin (Rapa, 100 ng/ml) for 2 h, and further stimulated with or without IGF-1 (10 ng/ml) for 10 h, followed by Western blotting with indicated antibodies.

Both rictor and mSin1 are the components of mTORC2 [1, 2], and S6K1 has recently been identified as the kinase that phosphorylates rictor (Thr1135) [37, 46, 47]. Particularly, it has been described that mSin1 can be phosphorylated on T86 and T398 by S6K1 in a cellular context-dependent manner [48]. Therefore, at the very beginning, we hypothesized that rapamycin may inhibit mSin1 phosphorylation by suppressing the activity of S6K1. To test this hypothesis, Rh30 cells were infected with recombinant adenovirus encoding constitutively active and rapamycin-resistant HA-tagged S6K1 mutant (F5A-E389-R3A) (Ad-S6K1-ca) and the control virus encoding GFP (Ad-GFP), respectively. In agreement with our previous data [49], expression of constitutively active and rapamycin-resistant (HA-S6K1-ca), but not GFP, increased the basal phosphorylation level of S6 ribosomal protein, a substrate of S6K1, and conferred resistance to rapamycin inhibition of S6 phosphorylation (Figure 4A), suggesting that the S6K1-ca was functional in the cells. However, expression of the rapamycin-resistant constitutively active S6K1 failed to prevent rapamycin from inhibiting the phosphorylation of mSin1 (Figure 4A). The results suggest that rapamycin-induced dephosphorylation of mSin1 is probably not by inhibiting S6K1 activity.

To corroborate the above finding, S6K1 was silenced using lentiviral shRNA to S6K1. We found that infection with lentiviral shRNA to S6K1, but not the control shRNA to GFP, downregulated the protein level of S6K1 by ~85% (vs. control), and abolished the basal or IGF-1-stimulated phosphorylation of S6K1, in Rh30 cells (Figure 4B), indicating the shRNA was working well in the cells. However, silencing S6K1 failed to reduce the basal or IGF-1-stimulated phosphorylation of mSin1, when rapamycin was absent (Figure 4B). The results support the notion that rapamycin-induced dephosphorylation (band shift) of mSin1 is independent of S6K1.

### Rapamycin inhibits phosphorylation of mSin1 independently of mTORC1

**Figure 5:**
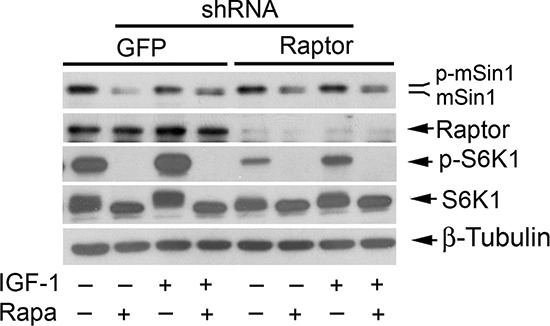
Rapamycin inhibits phosphorylation of mSin1 independently of mTORC1 Rh30 cells, infected with lentiviral shRNAs to GFP and raptor, respectively, were serum-starved for 24 h. The cells were then pretreated with or without rapamycin (Rapa, 100 ng/ml) for 2 h, and further stimulated with or without IGF-1 (10 ng/ml) for 10 h, followed by Western blotting with indicated antibodies.

mTORC1 is sensitive to rapamycin [1, 2]. It is likely that rapamycin inhibits mSin1 phosphorylation by targeting other mTORC1-mediated signaling molecules, instead of S6K1. For this, we directly determined whether mTORC1 is responsible for the phosphorylation of mSin1. In consistence with our previous findings [20, 49], infection with lentiviral shRNA to raptor, but not the control shRNA to GFP, downregulated the protein level of raptor by ~90% (vs. control) in Rh30 cells. Downregulation of raptor inhibited mTORC1-mediated phosphorylation of S6K1 (Thr389), a surrogate for mTORC1 activity in the cells (Figure 5). However, downregulation of raptor did not inhibit the basal or IGF-1-stimulated phosphorylation of mSin1 as rapamycin did (Figure 5). The results demonstrate that rapamycin inhibits mSin1 phosphorylation, which is independent of mTORC1.

### Rapamycin inhibits mSin1 phosphorylation independently of mTORC2

**Figure 6:**
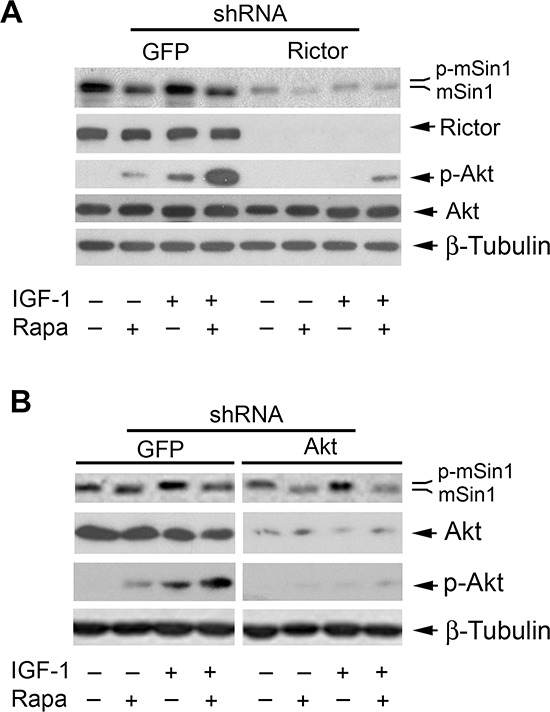
Rapamycin inhibits phosphorylation of mSin1 independently of mTORC2 Rh30 cells, infected with lentiviral shRNAs to GFP and rictor **(A)** or GFP and Akt **(B)** respectively, were serum-starved for 24 h. The cells were then pretreated with or without rapamycin (Rapa, 100 ng/ml) for 2 h, and further stimulated with or without IGF-1 (10 ng/ml) for 10 h, followed by Western blotting with indicated antibodies.

Currently, only two mTOR complexes, mTORC1 and mTORC2, have been identified [1, 2]. Since rapamycin-induced dephosphorylation of mSin1 was found to be independent of mTORC1 (Figure 5), next we turned to ask whether mTORC2 is involved in this event. To this end, mTORC2 was disrupted by silencing rictor. We found that infection with lentiviral shRNA to rictor, but not the control shRNA to GFP, downregulated the protein level of rictor by ~90% (vs. control) in Rh30 cells. Downregulation of rictor inhibited the basal or IGF-1 stimulated phosphorylation of Akt (Ser473) in the cells (Figure 6A). In line with our previous results [20], rapamycin induced phosphorylation of Akt (Ser473) in Rh30 cells (Figure 6A), although it inhibited phosphorylation of S6K1 and 4E-BP1 (Figure 2). As rictor and mSin1 interact with and stabilize each other [12], downregulation of rictor also reduced the cellular protein level of mSin1 correspondingly (Figure 6A). However, downregulation of rictor did not inhibit the basal or IGF-1-stimulated phosphorylation of mSin1 as rapamycin did (Figure 6A). Moreover, silencing Akt, a major substrate of mTORC2, did not reduce phosphorylation of mSin1 either (Figure 6B). The results indicate that rapamycin inhibits mSin1 phosphorylation independently of mTORC2 as well.

### mLST8 is essential for mTOR-mediated phosphorylation of mSin1

**Figure 7:**
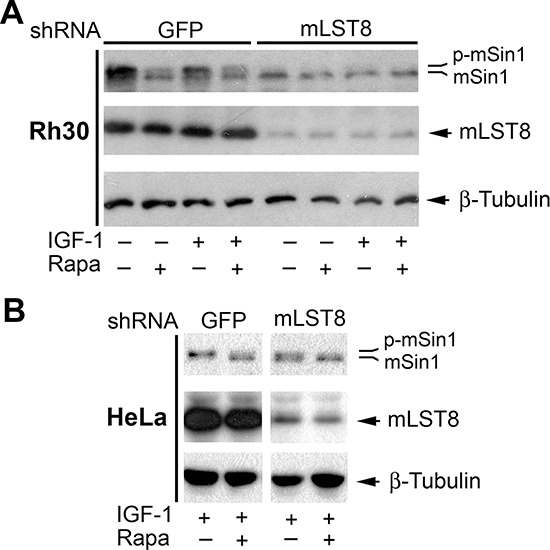
mLST8 is essential for mTOR-mediated phosphorylation of mSin1 Rh30 **(A)** or HeLa cells **(B)** infected with lentiviral shRNAs to GFP and mLST8, respectively, were serum-starved for 24 h. The cells were then pretreated with or without rapamycin (Rapa, 100 ng/ml) for 2 h, and further stimulated with or without IGF-1 (10 ng/ml) for 10 h, followed by Western blotting with indicated antibodies.

As mLST8 is an essential component for all mTOR complexes identified so far [1, 2], next we investigated whether the phosphorylation of mSin1 requires the involvement of mLST8. For this, Rh30 cells were infected with lentiviral shRNA to mLST8 or GFP (as a control). As illustrated in Figure 7A, mLST8 was downregulated by ~80%, in Rh30 cells by the lentiviral shRNA to mLST8. Silencing mLST8, like silencing mTOR (Figure 3B), inhibited the basal and IGF-1-stimulated phosphorylation of mSin1, even in the absence of rapamycin. Similar results were observed in HeLa cells (Figure 7B). The results indicate that mLST8 is necessary for mTOR-mediated phosphorylation of mSin1.

## DISCUSSION

mTOR functions as two complexes, mTORC1 and mTORC2, regulating cell growth, proliferation, survival, differentiation and motility [1, 2]. While intensive studies have focused on mTORC1, the mTORC2 signaling is only at the beginning to be recognized. Rictor and mSin1 interact with and stabilize each other, and are crucial for the integrity and the function of mTORC2 [12]. In addition to inhibiting mTORC1-mediated phosphorylation of S6K1 and 4E-BP1, rapamycin has recently been found to inhibit phosphorylation of rictor [36, 37] and mSin1 as well [38, 39]. Studies have revealed that S6K1 phosphorylates rictor (Thr1135), and rapamycin inhibition of rictor (Thr1135) phosphorylation is through suppressing S6K1 [37, 46, 47]. However, so far, how rapamycin inhibits mSin1 phosphorylation remains largely unknown. Here we show that rapamycin inhibited mSin1 phosphorylation in an mTOR kinase activity-dependent manner. This is supported by the observations that 1) expression of rapamycin-resistant kinase active mTOR (mTOR-T), but not rapamycin-resistant kinase dead mTOR (mTOR-TE), prevented rapamycin inhibition of mSin1 phosphorylation; and 2) silencing mTOR also inhibited mSin1 phosphorylation. Our findings strongly support the notion that mTOR regulates phosphorylation of mSin1.

To elucidate the molecular mechanism whereby rapamycin inhibits mSin1 phosphorylation, at the very beginning, we were focusing on S6K1, since S6K1 has been found to phosphorylate mSin1 on Thr86 and Thr398 [48]. However, in this study, we found that S6K1, in spite of being highly sensitive to rapamycin, was not involved in rapamycin-induced dephosphorylation of mSin1. This is evidenced by the findings that ectopic expression of rapamycin-resistant and constitutively active S6K1 (S6K1-ca) did not confer obvious resistance to rapamycin-induced dephosphorylation of mSin1, and silencing S6K1 did not induce the electrophoretic mobility of mSin1 as rapamycin did.

As rapamycin inhibited mSin1 phosphorylation within 2 h (Figure 2B), and mTORC1 is sensitive to acute rapamycin treatment [1, 2], we next reasoned that rapamycin-induced dephosphorylation of mSin1 is most likely by targeting mTORC1, although mTOR-mediated S6K1 pathway had been ruled out in this event (Figure 4). It has been described that rapamycin inhibits mTORC1, resulting in activation protein phosphatase 2A (PP2A) [50]. Possibly, rapamycin inhibits phosphorylation of mSin1 by activation of PP2A. However, out of our expectation, disruption of mTORC1 by silencing raptor did not inhibit mSin1 phosphorylation as rapamycin did (Figure 5), pointing out that the rapamycin-induced dephosphorylation of mSin1 is not mediated by mTORC1.

Although S6K1-mediated phosphorylation of rictor (Thr1135) does not affect mTORC2 integrity or *in vitro* kinase activity, it causes an increase in 14-3-3 binding to rictor and mTORC2-dependent phosphorylation of Akt (Ser473) in cells [37, 46, 47], suggesting a potential crosstalk between mTORC1 and mTORC2. After demonstrating that mSin1 phosphorylation is independent of mTORC1, we further examined whether this is dependent on mTORC2, although mTORC2 is only sensitive to prolonged rapamycin treatment in certain cases [18]. However, surprisingly, disruption of mTORC2 by silencing rictor failed to induce the electrophoretic mobility of mSin1 as rapamycin did, suggesting that rapamycin-induced dephosphorylation of mSin1 is independent of mTORC2 as well. This is further supported by the observation that downregulation of Akt, a major substrate of mTORC2, did not induce the electrophoretic mobility of mSin1 as rapamycin did. However, interestingly, silencing mTOR or mLST8 (a component shared by all mTOR complexes) recapitulated the effect of rapamycin on the phosphorylation of mSin1. Our results imply that rapamycin inhibits mSin1 phosphorylation possibly through targeting an unidentified third mTOR complex, which contains at least mTOR and mLST8. Hall group has reported that in addition to TORC1 and TORC2, actually TOR can interact with numerous proteins, as a 2-megadalton TOR protein complex has been detected by gel filtration [7]. Recently, a new TOR complex, which contains TOR, LST8 and Armadillo domain-containing protein (TbArmtor), has been identified in the parasite, *Trypanosoma brucei* [52]. Further research is needed to unveil whether there exists a similar counterpart in mammalian cells, and whether it mediates mSin1 phosphorylation.

Recently, it has been found that mSin1 can be phosphorylated on Thr86 and Thr398 by either Akt or S6K in a cell-dependent manner [48, 51]. Interestingly, the phosphorylation of mSin1 on both Thr86 and Thr398, but not on Thr86 alone, is required for the integrity and the activity of mTORC2 [48, 51]. In the present study, we found that downregulation of S6K1 or Akt failed to increase the electrophoretic mobility (dephosphorylation) of mSin1 under our experimental conditions, suggesting that rapamycin induced the band shift of mSin1 possibly by inducing dephosphorylation of more other residues. In consistence with our previous observation [20], here we found that rapamycin induced phosphorylation of Akt (Ser473) in Rh30 cells, but inhibited phosphorylation of mSin1 (Figure 6). Our results indicate that the Akt phosphorylation may be independent of rapamycin-induced dephosphorylation of mSin1.

mTOR is regulated by multiple upstream factors including growth factors and nutrients such as glucose and amino acids [1, 2]. In response to growth factors deprivation or nutrients starvation, mTORC1 can be inhibited, which may lead to reduced protein/lipid synthesis, inhibiting cell growth and inducing autophagy [1, 2]. It has been demonstrated that the small GTPase Rheb (Ras homolog enriched in brain) directly interacts with and activates mTOR [53]. Tuberous sclerosis complex 1/2 (TSC1/2), a tumor suppressor, acts as a GTPase-activating protein (GAP) for Rheb [54–57]. Growth factors or hormones (e.g. IGF-1 and insulin) activate mTORC1 by inhibiting TSC1/2 function [1, 2]. Hence, in response to growth factors deprivation or serum-starvation, mTORC1 activity is reduced in normal cells, but remains constitutively active in TSC-mutant cells [30]. Under glucose starvation (low ATP) condition, AMP-activated protein kinase (AMPK) can be activated [58]. Activated AMPK can inhibit mTORC1 signaling via activation of TSC1/2 and induce phosphorylation of raptor (Ser792) [59, 60]. Recently, it has been found that Rag small GTPases relay signals from amino acids to activate mTORC1 [61–63]. Amino acid starvation inhibits Rag-Ragulator mediated translocation of mTORC1 to lysosomal membranes, preventing mTORC1 activation [30].

In the present study, we noticed that serum-starvation or stimulation with IGF-1 did not affect the electrophoretic mobility of mSin1 (Figure 1A and 1B), but IGF-1 was found to slightly increase phosphorylation of mSin1 by ^32^P-labeling (Figure 1C). Rapamycin was able to increase the electrophoretic mobility of mSin1 in a spectrum of cell lines (Rh1, Rh30, PC-3, HeLa and MEF), regardless of presence or absence of IGF-1 (Figure 1A and 1B). The results suggest that only one or a few residues on mSin1 are sensitive to IGF-1-stimulated phosphorylation, but more residues are independent of growth factors for mTOR-mediated phosphorylation. It is known that mTORC1 is sensitive to rapamycin, growth factors, energy, amino acids, stress and redox levels [1, 2], whereas mTORC2 is only sensitive to prolonged (>24 h) rapamycin exposure in certain cases and growth factors [18]. Our observation implies that the unidentified third mTOR complex may be different from mTORC1 or mTORC2, which is sensitive to rapamycin, but may be insensitive to growth factors. Further research is required to address this issue. Recently, it has been described that mTOR stabilizes mSin1 by phosphorylating its hydrophobic and conserved Ser260 site to maintain the integrity of mTORC2 [64]. Also, glucose deprivation or acute ATP depletion, but not amino acid deprivation, prevents the mTOR-dependent phosphorylation of mSin1 on Ser260 and Akt on Thr450 in cells [64], further suggesting that mTOR may regulate phosphorylation of mSin1 on unique residues, in response to specific environmental cues. Therefore, it would be of great importance to unveil whether the new mTOR complex is sensitive to energy (glucose or ATP), amino acids, stress and redox levels, and whether it is evolutionally conserved from yeast to mammals.

**Figure 8:**
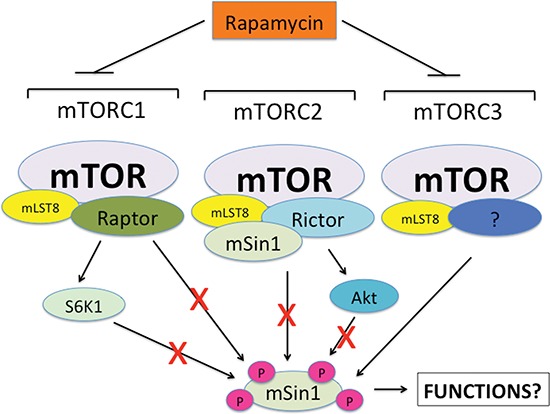
A proposed model showing how rapamycin inhibits mSin1 phosphorylation Neither mTORC1/S6K1 nor mTORC2/Akt was found to be involved in the regulation of mSin1 phosphorylation. However, silencing mTOR or mLST8 inhibited the phosphorylation of mSin1 as rapamycin did. Thus, we propose that rapamycin may inhibit mSin1 phosphorylation by targeting an unidentified third mTOR complex (mTORC3) that contains at least mTOR and mLST8.

In summary, here, for the first time, we have shown that rapamycin inhibited mSin1 phosphorylation, which were not through inhibiting either mTORC1/S6K1 or mTORC2/Akt pathway (Figure 8). However, silencing mTOR or mLST8 was able to inhibit the phosphorylation of mSin1 as rapamycin did. Our findings suggest that rapamycin may inhibit mSin1 phosphorylation probably by targeting an unidentified third mTOR complex (mTORC3) that contains at least mTOR and mLST8 (Figure 8). Further efforts are needed to identify this new complex, and to determine the phosphorylation site(s) of mSin1 as well as the physiological significance of the mSin1 phosphorylation.

## MATERIALS AND METHODS

### Materials

Rapamycin (LC Laboratories, Woburn, MA) was dissolved in dimethyl sulfoxide (DMSO) to prepare a 100 μg/ml stock solution and stored at −20°C. IGF-1 (PeproTech, Rocky Hill, NJ) was rehydrated in 0.1 M acetic acid to prepare a 10 μg/ml stock solution and stored at −80°C. Shrimp alkaline phosphatase (1,000 units/ml, New England BioLabs, Ipswich, MA). Enhanced chemiluminescence solution was from Pierce (Rockford, IL). Antibodies included those against mTOR, S6K1, Akt, S6, HA (Santa Cruz Biotechnology, Santa Cruz, CA), phospho-S6K1 (Thr389), phospho-Akt (Ser473), phospho-S6 (Ser/235/236), 4E-BP1 (Cell Signaling, Beverly, MA), raptor, rictor (Bethyl Laboratories, Montgomery, TX), mLST8 (GenWay Biotech, San Diego, CA), mSin1 (for Western blotting, K87 [13]; for immunoprecipitation, sc-48588, Santa Cruz Biotechnology), β-tubulin, FLAG (Sigma, St. Louis, MO); goat anti-rabbit IgG-horseradish peroxidase (HRP), goat anti-mouse IgG-HRP, rabbit anti-goat IgG-HRP, and goat anti-chicken IgG-HRP (Pierce). All other chemicals were purchased from Sigma.

### Cell culture

Human rhabdomyosarcoma Rh30 and Ewing sarcoma Rh1 cells were generously provided by Dr. Peter J. Houghton (Nationwide Children's Hospital, Columbus, OH), and grown in antibiotic-free RPMI 1640 medium (Mediatech, Herndon, VA) supplemented with 10% fetal bovine serum (FBS) (Hyclone, Logan, UT). Human cervical adenocarcinoma (HeLa), prostate adenocarcinoma (PC-3) (American Type Culture Collection, Manassas, VA), and wild-type (wt) mouse embryonic fibroblasts (MEF) [13] were grown in antibiotic-free Dulbecco's Modified Eagle Medium (DMEM) (Mediatech) supplemented with 10% FBS. Human embryonic kidney (HEK) 293 (American Type Culture Collection), 293A, and 293T cells (Invitrogen, Carlsbad, CA) were grown in antibiotic-free DMEM supplemented with 10% heat-inactivated FBS. All cell lines were grown in a humidified incubator at 37°C in an atmosphere of 5% CO_2_. For experiments where cells were deprived of serum, cell monolayers were washed with phosphate buffered saline (PBS), and incubated in the serum-free DMEM for at least 24 h.

### Recombinant adenoviral constructs and infection of cells

The recombinant adenoviruses expressing the green fluorescence protein (GFP) (Ad-GFP), FLAG-tagged rapamycin-resistant and kinase active mTOR (S2035T, mTOR-T) (Ad-mTOR-T), rapamycin-resistant and kinase dead mTOR (S2035T/D2357E, mTOR-TE) (Ad-mTOR-TE), and HA-tagged rapamycin-resistant and constitutively active mutant of S6K1 (F5A-E389-R3A) (Ad-S6K1-ca) were generated as described previously [20, 21, 49]. All adenoviruses were amplified, titrated and used as described [49].

### Lentiviral shRNA constructs and infection of cells

To generate lentiviral shRNA to human Akt1, oligonucleotides containing the target sequences were synthesized, annealed and inserted into FSIPPW lentiviral vector via the EcoR1/BamH1 restriction enzyme site. Oligonucleotides used were: sense, 5′-AATTCCCCGTGAGGCTCCCCTCAACATGCAAGA GATGTTGAGG GGAGCCTCACGTTTTTG-3′, and antisense, GATCCAAAAACGTGAGGCTCCCCTCA ACATCTCTTGCATGTTGAGGGGAGCCTCACGGGG-3′. Lentiviral shRNAs to GFP, mTOR, raptor, rictor and S6K1 were described previously [20]. Lentiviruses expressing indicated shRNAs were produced and titrated as described [49]. Cells, when grown to ~70% confluence, were infected with the above lentiviral shRNAs in the presence of 8 μg/ml polybrene and exposed to 2 μg/ml puromycin after 24 h of infection. In 5 days, cells were used for experiments.

### Western blotting

Cells were seeded in 6-well plates in appropriate medium containing 10% FBS. Next day, the cells were serum-starved in DMEM for 24 h, and then treated with or without rapamycin (100 ng/ml) for indicated time, followed by stimulation with or without IGF-1 (10 ng/ml) for indicated time. Western blotting was performed using indicated antibodies, as described previously [49].

### Detection of mSin1 phosphorylation by ^32^P labeling

Cells, grown in 100-mm dishes, were pretreated with or without rapamycin (100 ng/ml) for 24 h. Subsequently, the used medium was replaced with 3 ml phosphate-free DMEM (Invitrogen), and the cells were then stimulated with or without IGF-1 (10 ng/ml) for 1 h, followed by labeling with [^32^P] orthophosphate (0.4 mCi/ml) (MB Biomedicals, Solon, OH) for 4 h at 37°C. The cells were finally lysed in RIPA buffer [50 mM Tris, pH 7.2; 150 mM NaCl; 1% sodium deoxycholate; 0.1% SDS; 1% Triton-X 100; 10 mM NaF; 1 mM Na_3_VO_4_; protease inhibitor cocktail (1:1000, Sigma)], followed by immunoprecipitation with antibodies to mSin1 (sc-48588, Santa Cruz Biotechnology). The immunoprecipitates were separated by SDS-PAGE and the gel was dried. Phosphorylation of mSin1 (^32^P-labeled mSin1) was detected by autoradiography. The bands were semi-quantified using NIH image J.

### Statistical analysis

Results were expressed as mean values ± standard error (mean ± S.E.). Statistical analysis was performed using student *t*-test or one-way analysis of variance (ANOVA) followed by post hoc Dunnett's test for multiple comparisons. A level of *P* < 0.05 was considered to be significant.
