# Correction: Rapamycin inhibits mSin1 phosphorylation independently of mTORC1 and mTORC2

**DOI:** 10.18632/oncotarget.28745

**Published:** 2025-06-10

**Authors:** Yan Luo, Lei Liu, Yang Wu, Karnika Singh, Bing Su, Nan Zhang, Xiaowei Liu, Yangmei Shen, Shile Huang

**Affiliations:** ^1^State Key Laboratory of Biotherapy/Collaborative Innovation Center of Biotherapy, West China Hospital, Sichuan University, Chengdu, Sichuan, People’s Republic of China; ^2^Department of Biochemistry and Molecular Biology, Louisiana State University Health Sciences Center, Shreveport, LA, USA; ^3^Feist-Weiller Cancer Center, Louisiana State University Health Sciences Center, Shreveport, LA, USA; ^4^Department of Immunobiology, Yale University School of Medicine, New Haven, CT, USA; ^*^These authors have contributed equally to this work

**This article has been corrected:** Following an investigation into concerns raised by a third party, we have identified image panel duplication involving the western blot for S6K1 and mSin1 blot in Figure 3B. Additionally, the GFP portion of the S6K1 blot in Figure 3B was duplicated to create a portion of the S6K1 blot in Figure 4B. The authors have agreed with the identified issues and stated that duplication of the S6K1 blot was inadvertently introduced during the preparation of Figures 3B and 4B for publication. The authors provided the raw, uncropped blots for mSin1 (related to Figure 3B) and S6K1 (related to Figure 4B) from their original experiments. They have also prepared corrected versions of Figures 3 and 4, where the mSin1 blot in Figure 3B and the GFP part of the S6K1 blot in Figure 4B have been replaced with the correct blots. The authors have stated that these corrections do not alter the original results or conclusions of the paper and apologized for any inconvenience caused. The corrected figures are listed below.

Original article: Oncotarget. 2015; 6:4286–4298. 4286-4298. https://doi.org/10.18632/oncotarget.3006

**Figure 3:**
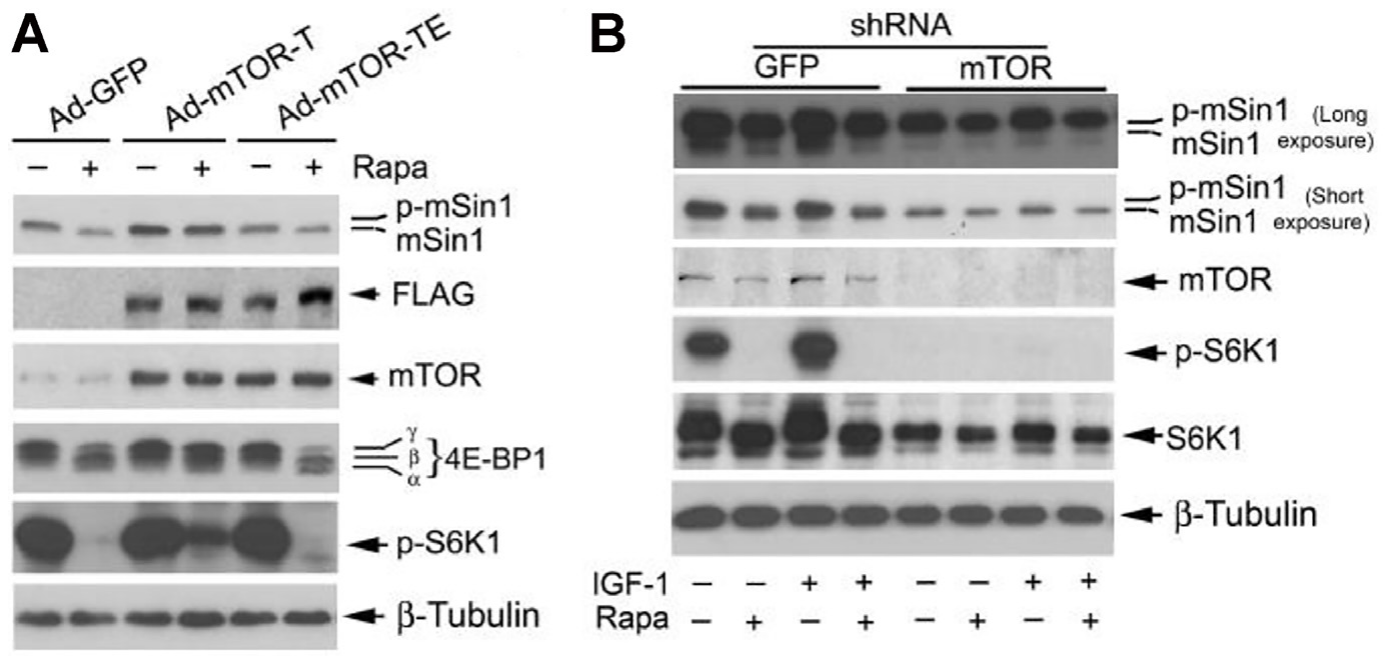
Rapamycin-induced dephosphorylation of mSin1 is dependent on mTOR kinase activity. Rh30 cells, infected with recombinant adenoviruses expressing GFP (Ad-GFP), FLAG-tagged rapamycin-resistant and kinase active mTOR (S2035T, Ad-mTOR-T), and rapamycin-resistant and kinase dead mTOR (S2035T/D2357E, Ad-mTOR-TE) (**A**) or with lentiviral shRNAs to GFP and mTOR (**B**) respectively, were serum-starved for 24 h. The cells were then pretreated with or without rapamycin (Rapa, 100 ng/ml) for 2 h, and further stimulated with or without IGF-1 (10 ng/ml) for 10 h, followed by Western blotting with indicated antibodies.

**Figure 4:**
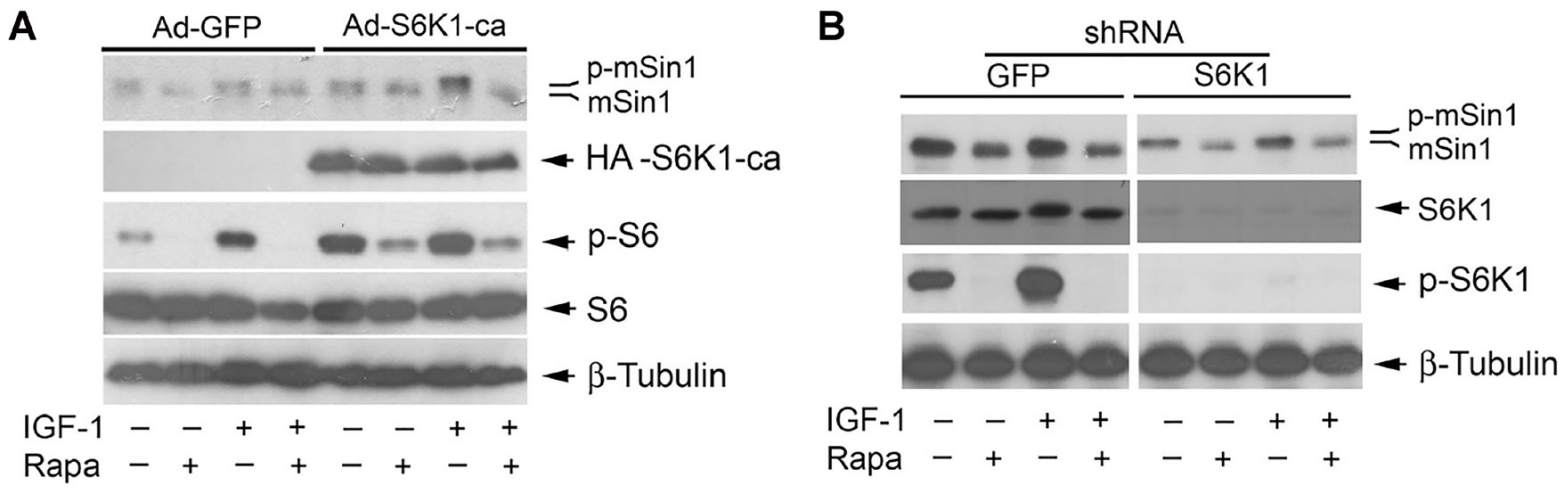
Rapamycin-induced dephosphorylation of mSin1 is not by inhibiting S6K1. Rh30 cells, infected with recombinant adenoviruses expressing GFP (Ad-GFP) and HA-tagged rapamycin-resistant and constitutively active S6K1 (Ad-S6K1-ca) (**A**) or with lentiviral shRNAs to GFP and S6K1 (**B**) respectively, were serum-starved for 24 h. The cells were then pretreated with or without rapamycin (Rapa, 100 ng/ml) for 2 h, and further stimulated with or without IGF-1 (10 ng/ml) for 10 h, followed by Western blotting with indicated antibodies.

